# Suppressive action of miRNAs to ARP2/3 complex reduces cell migration and proliferation *via* RAC isoforms in Hirschsprung disease

**DOI:** 10.1111/jcmm.12799

**Published:** 2016-03-16

**Authors:** Weibing Tang, Peng Cai, Weiwei Huo, Hongxing Li, Junwei Tang, Dongmei Zhu, Hua Xie, Pingfa Chen, Bo Hang, Shouyu Wang, Yankai Xia

**Affiliations:** ^1^ Department of Pediatric SurgeryNanjing Children's Hospital Affiliated Nanjing Medical UniversityNanjingChina; ^2^ State Key Laboratory of Reproductive MedicineInstitute of ToxicologySchool of Public HealthNanjing Medical UniversityNanjingChina; ^3^ Children's Hospital of Soochow UniversitySoochowChina; ^4^ Key Laboratory of Modern Toxicology (Nanjing Medical University)Ministry of EducationNanjingChina; ^5^ Department of Cell and Molecular BiologyLife Sciences DivisionLawrence Berkeley National LaboratoryBerkeleyCAUSA; ^6^ Department of Molecular Cell Biology and ToxicologyJiangsu Key Lab of Cancer Biomarkers, Prevention & TreatmentCancer CenterSchool of Public HealthNanjing Medical UniversityNanjingChina

**Keywords:** microRNA, ARP2/3 complex, RAC isoforms, gene regulation, Hirschsprung disease

## Abstract

Hirschsprung disease (HSCR) is a congenital disorder caused by the defective function of the embryonic enteric neural crest. The impaired migration of embryonic enteric neural crest plays an important role in the pathogenesis of this disease. Recent studies showed that the ARP2/3 complex and RAC isoforms had effects on actin cytoskeleton remodelling, which contributes to migration. Moreover, some regulatory relationships were identified between ARP2/3 complex and RAC isoforms. Although microRNAs (miRNAs) have been known to modulate target gene expression on the post‐transcriptional level, little is known about the regulation among miRNAs, ARP2/3 complex and RAC isoforms. Here, we report that down‐regulation of ARP2 and ARP3, two main subunits of ARP2/3 complex, suppressed migration and proliferation in 293T and SH‐SY5Y cell lines *via* the inhibition of RAC1 and RAC2. Meanwhile, as the target genes, ARP2 and ARP3 are reduced by increased miR‐24‐1* and let‐7a*, respectively, in 70 HSCR samples as compared with 74 normal controls. Co‐immunoprecipitation showed that aberrant reduction in ARP2 and ARP3 could weaken the function of ARP2/3 complex. Our study demonstrates that the miR‐24‐1*/let‐7a*‐ARP2/3 complex‐RAC isoforms pathway may represent a novel pathogenic mechanism for HSCR.

## Introduction

Hirschsprung disease (HSCR) is a congenital abnormality disorder and is caused by the absence of ganglion cells (aganglionosis) in a variable length of the gastrointestinal tract, with an incidence of approximately 1 in 5000 births 1. It is characterized by the defective migration of the enteric neural crest cells (ENCCs) and their failed colonization to the distal bowel during embryogenesis from 5 to 12 weeks 2, 3. Because of a defective enteric nervous system (ENS) 3, 4, either a few centimetres of the gut near the anus or the entire colon loses intestinal motility, thus leading to constipation, abdominal distension, bilious vomiting, growth failure and life‐threatening infection 5, 6. According to aetiologic studies, both genetic factors and environmental factors can result in HSCR. Many genes, especially *RET*, have been identified to be associated with the pathogenesis of HSCR 3, 4, 7. Polygenic abnormal expression attributes to a failure of cell migration, which is part of HSCR pathogenesis. Some studies have also shown the importance of the actin cytoskeleton for migration of ENCCs, which suggests another pathogenic factor for HSCR 8.

The actin cytoskeleton has important roles in a wide range of cellular processes, such as motility and pseudopod movement. Various factors contribute to the regulation of actin, including the actin‐related protein 2/3 (ARP2/3) complex. As a branched actin nucleator, it consists of seven subunits, including two main subunits (ARP2 and ARP3) and other five subunits (ARPC1‐5) that provide a framework for positioning the dimer formed by ARP2 and ARP3 9. The complex binds existing actin mother filaments and promotes the formation of daughter filaments, the latter are key to the nucleation of this branched actin network 10. To keep the full activity of ARP2/3 complex, nucleation promoting factors are needed, such as Wiskott–Aldrich syndrome protein (WASP), N‐WASP, WAVEs, WASH and WHAMM 10, 11. In some previous studies, Arp2/3 complex could act with Rho guanosine triphosphatases (GTPases), the downstream receptors of the heptamer 12. In mammals, the Rho family of GTPases is composed of more than 20 members, which belongs to the Ras superfamily 13. According to the domain structure and function, these members can be subdivided into five subfamilies, RAC, Rho, Cdc42, Rho‐BTB and Rnd 13. Most Rho GTPases stimulate a variety of cellular processes, such as migration, cell adhesion, pathway signalling during cytokinesis, neuronal development and reorganization of the actin cytoskeleton 14, 15, 16. As one subfamily of Rho GTPases, RAC subfamily includes four members, RAC1, RAC2, RAC3 and RhoG. RAC1, 2 and 3 have higher sequence similarity among them (more than 90%) than with RhoG (72%, comparing with RAC1) 17, but their expression levels and locations are different. RAC1 and RhoG are widely expressed, whereas RAC2 expression is mostly restricted to cells of haematopoietic origin 18 and RAC3 is highly expressed in the central nervous system 19. According to multiple studies, the RAC isoforms are involved in the branched actin network by regulating ARP2/3 complex in RAC‐WAVE/Arpin‐ARP2/3 complex pathway 20. In addition, previous studies demonstrate that RAC acts downstream of ARP2/3 complex 12 and that a feed‐back loop exists among ARP2/3 heptamer, Tiam1 and RAC 21. Given the significance of the ARP2/3 complex, we carried out the studies in this work, as we found little evidence supporting the relationship between ARP2/3 complex and microRNAs (miRNAs).

MiRNAs are small, non‐coding RNA molecules, which act as repressors in post‐transcriptional control. They are about 19–25 nucleotides long and negatively regulate their target genes by base‐pairing with the complementary sequences in the 3′‐untranslated regions (3′‐UTR) of the target mRNAs 22. MiRNA‐involved regulatory networks are involved in various cellular processes, including cell cycle, migration, apoptosis and synaptic plasticity 23, 24, 25. For example, overexpression of miR‐28‐5p reduced cell proliferation and migration in colorectal cancer 26. Other examples include that miR‐206 regulates cell proliferation kinetics 27 and miR‐141 contributes to migration and proliferation 28. Based on such studies, we suggested that certain miRNAs act as potential upstream regulators of the ARP2/3 complex. Indeed, this work has revealed a novel mechanism that is regulated by miRNAs through ARP2/3 complex and RAC isoforms in the pathogenic process of HSCR.

## Materials and methods

### Ethics statement and tissues samples

This research was approved by the Institutional Ethics Committee of Nanjing Medical University. All research activities involving human cases were carried out in accordance with government policies and the Helsinki Declaration. 70 HSCR patients with a pathological diagnosis and 74 matched controls were selected. Colon tissues were collected from patients undergoing surgical treatment at Nanjing Children's Hospital Affiliated to Nanjing Medical University from October 2009 to April 2013 (the NJMU Birth Cohort). Tissues were collected with informed consent and agreement from patients and were stored at −80°C. All the patients were diagnosed by barium enema and anorectal manometry evaluation before surgical procedures. After surgery, pathological examination was taken to make a definite diagnosis. The control samples were collected after an enterectomy intussusception or inguinal hernia without the ischaemic or necrotic parts.

### RNA extraction and quantitative real‐time PCR (qRT‐PCR)

Total RNA was extracted from tissue samples and cell lines using Trizol reagent (Life technologies, Carlsbad, California) according to the manufacturer's instructions. The qRT‐PCR was carried out to measure the expression levels of miRNAs and mRNAs. For miRNAs, TaqMan^®^ MicroRNA Assays (Applied Biosystems, Carlsbad, California) were chosen for has‐miR‐24‐1* and has‐let‐7a*. Sn‐RNA U6 was used as a control for normalization. We used 1 μg total RNAs as the template and reverse transcription conditions were as follows: 16°C, 30 min., 42°C, 30 min. and 85°C, 5 min. The cDNAs were 1:40 diluted, and PCR was performed for 5 sec. at 95°C and for 30 sec. at 60°C, 40 cycles.

Glyceraldehyde‐3‐phosphate dehydrogenase (GAPDH) was used as an internal control for mRNAs. We extracted total RNAs (500 ng) under 37°C for 15 min. and 85°C for 30 sec. for reverse transcription using the reverse transcription kit (Takara, Tokyo, Japan). PCR amplification was performed in an ABI 7900 HT (Applied Biosystems 7900 HT, Carlsbad, California, U.S.A) under 5 sec. at 95°C and for 30 sec. at 60°C for 40 cycles. All the primers were shown in Table 1.

**Table 1:** Primers of genes mentioned in the study

Gene	Forward primer	Reverse primer
ARP2	5′‐GGCAGTTCTGACTTTGTACGC‐3′	5′‐CCAGTCTCCTGGTAAGATGAGG‐3′
ARP3	5′‐TTGAGTGGTGGTAGATTGAAGC‐3′	5′‐CCAAACTGCATATCGCTGCAT‐3′
RAC1	5′‐ATGTCCGTGCAAAGTGGTATC‐3′	5′‐CTCGGATCGCTTCGTCAAACA‐3′
RAC2	5′‐CAACGCCTTTCCCGGAGAG‐3′	5′‐TCCGTCTGTGGATAGGAGAGC‐3′
RAC3	5′‐AATTCATGCAGGCCATCAAGT‐3′	5′‐CTAGAAGACGGTGCACTT‐3′
GAPDH	5′‐GCACCGTCAAGGCTGAGAAC‐3′	5′‐GGATCTCGCTCCTGGAAGATG‐3′

### Western blot analysis

Total proteins were collected from tissue samples and treated cell lines with RIPA buffer containing protease inhibitors cOmplete, ULTRA, Mini, EDTA‐free, EASYpack (Roche, Basel, Switzerland). The BCA method was used to determine protein concentrations. Proteins were subjected to 12% SDS‐PAGE and then transferred onto a nitrocellulose membrane. Membrane was blocked using 5% skimmed milk and incubated with antibodies. Primary antibodies of ARP2 (sc15389), ARP3 (sc15390), RAC1 (sc95) and RAC2 (sc96) were from Santa Cruz (Santa Cruz Biotechnology, Santa Cruz, CA, USA) and RAC3 (Cat. no.: 5659‐1) was from Epitomics, Inc. (Burlingame, CA, USA). The target proteins were detected using ECL reagent kit (Millpore, Boston, Massachusetts, U.S.A). A GAPDH antibody (Beyotime, Nantong, China) was used for endogenous control.

### Cell culture and transfections

Human 293T and SH‐SY5Y cell lines were purchased from American Type Culture Collection (ATCC, Manassas, VA, USA), and were cultured in a complete growth medium DMEM (Hyclone, Thermo Fisher, Logan, Utah, U.S.A.) supplemented with 10% foetal bovine serum (10% FBS), 100 U/ml penicillin and 100 μg/ml streptomycin at 37°C, 5% CO_2_. Synthetic miRNAs of negative control, miR‐24‐1* and let‐7a*, and siRNAs of ARP2, ARP3, RAC1 and RAC2 (GenePharma, Shanghai, China) were used in cell transfection coupled with Lipofectamine 2000 Reagent (Invitrogen, Carlsbad, California) following the manufacturer's instructions. RNA oligos mentioned in this study were listed in Table 2.

**Table 2:** Related RNA oligos

Oligo	Sense	Anti‐sense
hsa‐miR‐24‐1* mimics	5′‐UGCCUACUGAGCUGAUAUCAGU‐3′	5′‐UGAUAUCAGCUCAGUAGGCAUU‐3′
has‐let‐7a* mimics	5′‐CUAUACAAUCUACUGUCUUUC‐3′	5′‐AAGACAGUAGAUUGUAUAGUU‐3′
siRNA‐ARP2	5′‐GGCACCGGGUUUGUGAAGUTT‐3′	5′‐ACUUCACAAACCCGGUGCCTT‐3′
siRNA‐ARP3	5′‐CCGCCAUGGUAUAGUUGAATT‐3′	5′‐UUCAACUAUACCAUGGCGGTT‐3′
siRNA‐RAC1	5′‐CUACUGUCUUUGACAAUUATT‐3′	5′‐UAAUUGUCAAAGACAGUAGTT‐3′
siRNA‐RAC2	5′‐CCACCGUGUUUGACAACUATT‐3′	5′‐UAGUUGUCAAACACGGUGGTT‐3′
Control	5′‐UUCUCCGAACGUGUCACGUTT‐3′	5′‐ACGUGACACGUUCGGAGAATT‐3′

### Transwell invasion assay

After transfection, the harvested cells were suspended in serum free medium (1 × 10^6^ cells/ml), 100 μl of which was added to the upper compartment of the chambers (8‐μm pore size, Millipore Corporation, Billerica, MA, USA). A conditioned medium with 10% (v/v) FBS was used as a chemoattractant and placed in the bottom compartment of the chamber. After 24 hrs of incubation, the invaded cells were stained with the crystal violet staining solution (Beyotime) for 15 min., and the cells were quantified from five different fields under 40× magnification (five views per well). All these experiments were replicated three times.

### Cell proliferation assay

Cell proliferation was determined by the Cell Counting Kit 8 (CCK‐8; Beyotime) after transfection for 24 hrs. 10^4^ cells/well in 100 μl were plated into a 96‐well plate after 48 hrs transfection, after incubation at 37°C for 4 hrs, the absorbance of samples was measured at 450 nm by TECAN infinite M200 Multimode microplate reader (Tecan, Mechelen, Belgium).

### Cell cycle and apoptosis analysis

BD Biosciences FACS Calibur Flow Cytometry (BD Biosciences., Franklin Lakes, New Jersey) and Annexin V‐FITC/Propidium Iodide Kit (KeyGen Biotech, Nanjing, China) were used for detection of apoptosis and cell cycle, respectively, and all the tests were repeated three times.

### Dual‐luciferase reporter assay

The 3′‐UTR sequences of ARP2 and ARP3, containing the putative target sites for miR‐24‐1* and let‐7a*, respectively, were inserted into the KpnI and SacI sites of pGL3 promoter vector (Genscript, Nanjing, China). The resulting constructs were named pGL3‐ARP2, pGL3‐ARP2‐mut, pGL3‐ARP3 and pGL3‐ARP3‐mut. Cells were plated in 24‐well plates and cotransfected with 100 ng of pGL3‐ARP2, pGL3‐ARP2‐mut and 50 nM miR‐24‐1* mimics and negative control using Lipofectamine 2000 (Invitrogen). For pGL3‐ARP3, pGL3‐ARP3‐mut and let‐7a* mimics, we performed the same procedure towards pGL3‐ARP2, pGL3‐ARP2‐mut and miR‐24‐1*. A renilla luciferase vector pRL‐SV40 (5 ng) was also cotransfected to normalize the difference in transfection efficiency. Luciferase activity levels after 48 hrs incubation were measured using a Dual‐Luciferase Reporter Assay System (Promega, Madison, WI, USA) according to manufacturer's instructions. Transfection was repeated in triplicate.

### Co‐immunoprecipitation

Cells were transfected for 24 hrs with siRNA‐ARP2 or siRNA‐ARP3 for co‐immunoprecipitation (co‐IP). Cells were harvested and lysed in cold RIPA Lysis Buffer (Beyotime) in the presence of 1 mM PMSF. The cell extracts were centrifuged at 10,000 × g at 4°C for 15 min., and supernatants were incubated with protein A/G agarose beads (Beyotime) or a control IgG (Beyotime). Cell lysates containing 1 mg of protein were incubated with 2 μg of anti‐ARP2 or anti‐ARP3 antibody at 4°C for 1 hr, followed by incubation overnight with protein A/G agarose beads. The immunoprecipitates were collected and washed twice with the lysis buffer. 5× SDS loading buffer was added and incubated at 95°C for 5 min. for SDS‐PAGE and Western blotting was subsequently performed with indicated antibodies.

### Statistical analysis

The data from the tissue samples in this study are presented as box plot of the median and range of log‐transformed relative expression level using Mann–Whitney *U*‐test, and the data obtained from the cell lines are presented as mean ± S.E.M. from three separate experiments, which are analysed by double‐sided Student's *t*‐test. *P* < 0.05 was considered statistically significant.

## Results

### Samples information analysis

Colon tissues taken from 70 patients pathologically diagnosed with HSCR were used for this study. Seventy‐four colon tissues from normal individuals were used as controls. The ages of patients with HSCR and of control groups were 3.12 ± 0.23 and 2.82 ± 0.20 months old respectively. The gender ratio was 54/16 and 61/13 (male/female), and the bws were 4.86 ± 0.10 and 4.75 ± 0.14 kg respectively. The 70 patients included in this study consisted of 33 short‐segment and 37 long‐segment. All the information demonstrated no statistical differences in the specimens obtained between HSCR patients and controls (Table 3).

**Table 3:** Demographic and clinical features of study cases

Variable	HSCR (*n* = 70)	Control (*n* = 74)	*P*
Age (months, mean, S.E.)	3.12 (0.23)	2.82 (0.20)	0.27^a^
Sex (%)			
Male	54 (77.14)	61 (82.43)	0.429^b^
Female	16 (22.86)	13 (17.57)	
Weight (kg, mean, S.E.)	4.86 (0.10)	4.75 (0.14)	0.58^a^
Classification (%)			
Short‐segment	33 (47.14)		
Long‐segment	37 (52.86)		

a Student *t*‐test.

b Two‐sided chi‐squared test.

### mRNA and protein expression levels of ARP2 and ARP3 were down‐regulated, whereas RAC isoforms showed differential expression in HSCR

**Figure 1:**
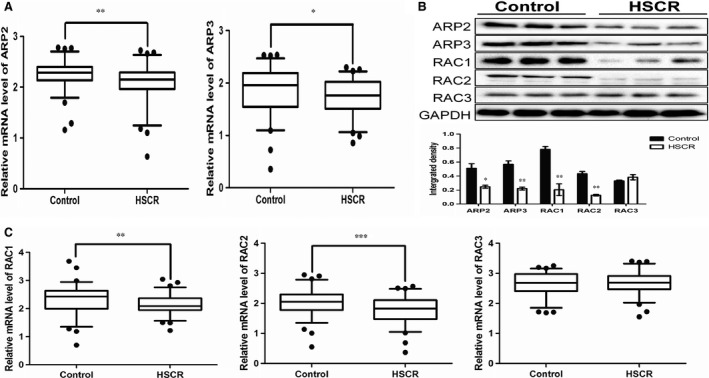
The expression level of ARP2, ARP3 and RAC isforms in HSCR/control tissues. (**A**) The mRNA expression level of ARP2 and ARP3 in control/HSCR tissues (**P = 0.0043, *P = 0.0256, *n* = 74 controls/70 HSCR, Mann–Whitney test). (**B**) The protein expression level of ARP2, ARP3, RAC1, RAC2 and RAC3 in HSCR/control tissues (*P < 0.05, **P < 0.01, *n* = 3, Unpaired *t*‐test). (**C**) The mRNA expression level of RAC1, RAC2 and RAC3 in control/HSCR tissues (**P = 0.0051, ***P = 0.0005, *n* = 74 controls/70 HSCR, Mann–Whitney test). All tests were performed for three times and presented as mean ± S.E.M.

TaqMan quantitative real‐time PCR was used to verify the relative expression levels of ARP2 and ARP3, with GAPDH being used to normalize the final qRT‐PCR data (Fig. 1A). The protein expression levels of ARP2 and ARP3 were also confirmed by Western blot (Fig. 1B). Data were analysed by the gray value quantified by ImageJ. These experiments indicated that both ARP2 and ARP3 were down‐regulated in this disease.

To investigate the possible relationship between the ARP2/3 complex and RAC isoforms, we examined the mRNA and protein expression levels of all RAC isoforms using the same approach as mentioned above (Fig. 1B and C). We observed that only RAC1 and RAC2 were down‐regulated with statistically significant differences. However, no significant difference in expression was observed for RAC3 in patients and controls.

### Co‐immunoprecipitation assay

**Figure 2:**
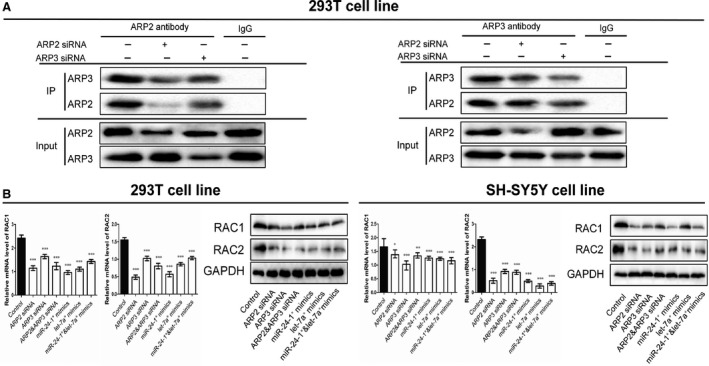
The CO‐IP results and RAC1/2 were inhibited by upstream regulators. (**A**) The co‐immunoprecipitation results of ARP2 and ARP3 in 293T cell line. The cells were pre‐treated with the siRNAs of ARP2 or ARP3 and endogenous protein–protein interaction between ARP2 and ARP3 was demonstrated by immunoprecipitation (IP) with ARP2 or ARP3 antibodies. IgG was used as negative control for IP. (**B**) The mRNA and protein expression levels of RAC1 and RAC2 after the transfection of siRNA‐ARP2, siRNA‐ARP3, miR‐24‐1* mimics and let‐7a* mimics in 293T and SH‐SY5Y cell lines (*P < 0.05, **P < 0.01, ***P < 0.001, *n* = 3, Unpaired *t*‐test). All tests were performed for three times and presented as mean ± S.E.M.

The ARP2/3 complex is composed of seven stoichiometric subunits, including two actin‐related proteins, ARP2 and ARP3, whose binding activity maintains the function of the whole complex. To determine whether the suppression of ARP2 and ARP3 may affect the binding, we performed co‐IP assay after transfection of siRNA‐ARP2 and siRNA‐ARP3 in human 293T cell line. As shown in Figure 2A, decreased ARP3 and ARP2 contributed to binding inhibition of ARP2 and ARP3 respectively, which could further contribute to block the formation of the ARP2/3 complex.

### Suppressed ARP2/3 inhibited RAC1 and RAC2 accompanied by decreased migration and proliferation

**Figure 3:**
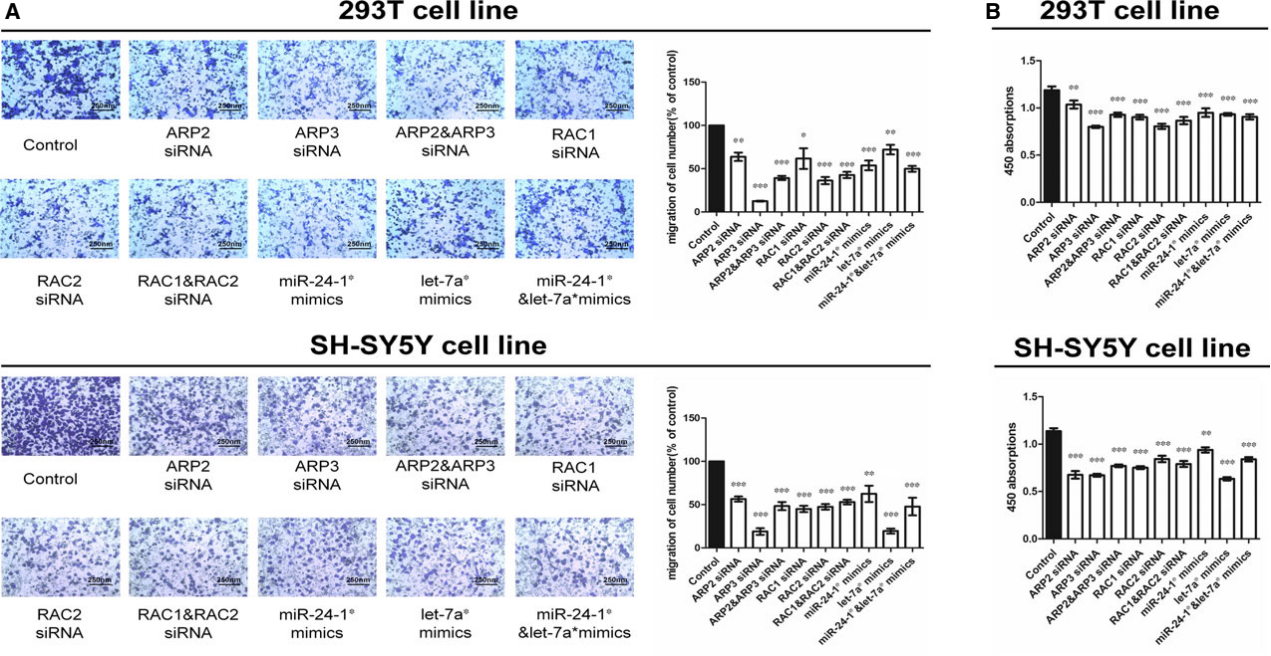
Cytobiology change after transfecting cell lines with RNA oligos. (**A**) The representative images of metastasis cells at the bottom of the membrane stained with crystal violet were visualized as shown (left). The quantifications of cell migration were presented as percentage migrated cell numbers and the integrated intensity of migrated cells (right) (*P < 0.05, **P < 0.01, ***P < 0.001, *n* = 5, Unpaired *t*‐test). (**B**) The result of CCK‐8 assay of 450 nm absorption (*P < 0.05, **P < 0.01, ***P < 0.001, *n* = 6, Unpaired *t*‐test). All tests were performed for three times and presented as mean ± S.E.M.

To ensure the function of ARP2 and ARP3, we transfected ARP2 and ARP3 siRNAs into human 293T and SH‐SY5Y cell lines. We have performed the experiments to show that the siRNA treatment had worked (Fig. S2). The data showed that down‐regulation of ARP2 and ARP3 resulted in reduced cell proliferation and migration (Fig. 3A and B), coupled with down‐expressed mRNA and protein levels of RAC1 and RAC2 (Fig. 2B). Moreover, we performed cotransfection of ARP2 and ARP3 siRNAs to identify a possible synergistic effect. When compared with the single ARP2 or ARP3 treatment, no obvious differences between the cotransfection group and the single transfection groups were observed in proliferation and migration, neither in the expression levels of RAC1 and RAC2 mRNAs and proteins.

### Down‐regulated RAC1 and RAC2 suppressed proliferation and migration

Based on the above data, we speculated that RAC1 and RAC2 might be associated with cell proliferation and migration. Therefore, we transfected siRNA‐RAC1 and siRNA‐RAC2 into the two human cell lines mentioned above. As expected RAC1 deficiency inhibited proliferation and migration *in vitro*, so did siRNA‐RAC2, which was consistent with our hypothesis above (Fig. 3A and B). Cotransfection of both siRNAs in the same cell lines was performed, but no synergistic effect was identified.

### miR‐24‐1* and let‐7a* are up‐regulated in HSCR and directly target ARP2 and ARP3 respectively

We used DIANA‐MICROT (http://diana.cslab.ece.ntua.gr/) and MICRORNA.ORG (http://www.microrna.org/) to backward predict the upstream regulators of ARP2 and ARP3. The results indicated that miR‐24‐1* and let‐7a* have an ilka combination with the 3′‐UTR of ARP2 and ARP3 mRNAs.

**Figure 4:**
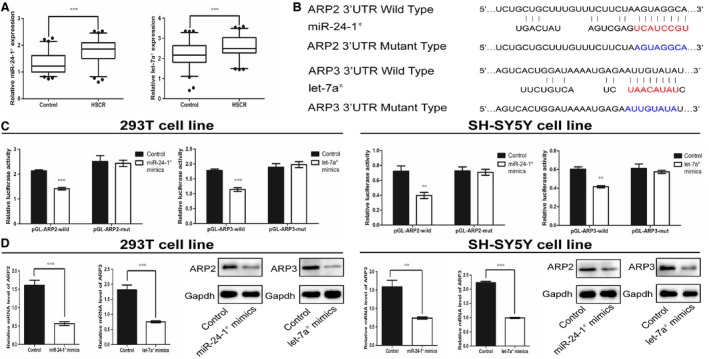
Up‐regulated miR‐24‐1* and let‐7a* respectively inhibited ARP2 and ARP3 in double cell lines. (**A**) The expression level of miR‐24‐1* and let‐7a* in HSCR/control tissues (***P ≤ 0.0003, *n* = 74 controls/70 HSCR, Mann–Whitney test). (**B**) The binding sequence of miR‐24‐1*&ARP2 wild‐type, let‐7a*& ARP3 wild‐type and the sequence of mutant type of ARP2 and ARP3 in 3′‐UTR. (**C**) The results of luciferase reporter assay (**P < 0.01, ***P < 0.001, *n* = 8, Mann–Whitney test). (**D**) The mRNA and protein expression level of ARP2 and ARP3 after ilka transfection of miR‐24‐1* and let‐7a* mimics in 293T and SH‐SY5Y cell lines (**P < 0.01, ***P < 0.001, *n* = 6, Unpaired *t*‐test). All tests were performed for three times and presented as mean ± S.E.M.

To identify the relative expression levels of miR‐24‐1* and let‐7a* in HSCR patients and controls, we performed TaqMan qRT‐PCR and the data were normalized to snRNA‐U6. Compared with control tissues, the expression levels of miR‐24‐1* and let‐7a* were higher in HSCR cases (Fig. 4A), which suggests that miR‐24‐1* and let‐7a* may contribute to the pathogenesis of HSCR by possible targeting ARP2 and ARP3 respectively.

Luciferase assay were performed to confirm this hypothesis. Compared with the control, overexpression of miR‐24‐1* and let‐7a* significantly suppressed luciferase activity of report genes containing 3′‐UTR of ARP2 and ARP3 respectively. However, such an effect was partially affected when the binding site of ARP2 or ARP3 was changed, suggesting that ARP2 and ARP3 are the ilka predicted target of miR‐24‐1* and let‐7a* respectively (Fig. 4B and C).

### Increased mir‐24‐1* and let‐7a* suppressed cell proliferation and migration through the miR‐24‐1*/let‐7a*‐ARP2/3 complex‐RAC isoforms pathway in HSCR

To demonstrate the regulatory relationship between miR‐24‐1*/let‐7a* and their respective target genes ARP2/ARP3, we up‐regulated the miR‐24‐1* and let‐7a* in 293T and SH‐SY5Y cell lines. To identify the synergistic action between miR‐24‐1* and let‐7a*, we performed the treatment with both miR‐24‐1* and let‐7a* mimics. The data showed that up‐regulation of miR‐24‐1* resulted in down‐regulation of ARP2 at both mRNA and protein levels, and likewise, let‐7a* mimics led to down‐regulation of ARP3 (Fig. 4D). Both mimics also down‐regulated RAC1 and RAC2 at both mRNA and protein levels (Fig. 2B). Based on the *in vitro* experiments shown above, both mimics of miR‐24‐1* and let‐7a* reduced cell proliferation and migration (Fig. 3A and B). In addition, no significant synergistic effect was observed in these experiments.

Apoptosis and cell cycle progression were determined using flow cytometry. Overexpression of miR‐24‐1* and let‐7a* did not lead to a significant difference in both cell cycle and apoptosis in HSCR patients when compared to the control group (Fig. S1).

## Discussion

The ENS originates from the neural crest cells, and the migration of ENCCs from the foregut to the rectum takes 7 weeks to occur in human embryonic development. During this process, the alteration of internal environment and aberrant expression of genes or molecules may contribute to aganglionosis 29. In general, the pathogenesis of HSCR concerns with the aberrant function of ENCCs 30. Many factors affect the function of ENCCs, such as changes of internal environment, aberrant expression of various cell components, nutritional status, abnormal activation of cell signalling and exogenous chemical exposure. Moreover, any factor that extraordinarily modulate the proliferation, apoptosis, cell cycle and autophagy of ENCCs may lead to a congenital megacolon.

The pathogenesis of HSCR contains different aspects, but one main reason underlying its development is the aberrant migration of ENCCs during embryogenesis. Current research on the disease has a variety of directions, including epigenetics, environmental chemical exposure, enteral environment and so on. An unpublished study in our group aims to investigate the single nucleotide polymorphisms in the patients with HSCR. The patients used in the detection covered the patients in this study. The Exome‐wide scan was performed by using the Illumina Human Exome Bead Chips. We analysed the genes which were reported to be associated with HSCR including RET, EDNRB, NRG1, SOX10, *etc*. We found that no significant difference was presented between the case and control group. Besides the classic disease‐causing genes, such as RET and SOX10, many other genes have been found to be associated to ENS development include Meis3, FOXA1, NTF‐3 31, 32, 33. Some chemicals, like Benzophenone‐3, was found to be involved in the pathogenesis of HSCR 34. Furthermore, studies about single nucleotide polymorphism also found some risk factors of HSCR 35, 36. Based on the study of non‐coding RNA, we found some novel pathways, include SLIT2/ROBO1‐miR‐218‐1‐RET/PLAG1, miR‐192/miR‐215/NID1 and miR‐200a/141/PTEN 37, 38, 39, which might control the migration of ENCCs. But it was the first time that we found subunits role of ARP complex in the pathogenesis of HSCR.

Based on the reported function of the ARP2/3 complex 40, we first verified the expression levels of ARP2 and ARP3 in HSCR colon samples, and found that they were down‐regulated in HSCR cases. To explore the genes that might interact with the heptamer, we focused on the RAC isoforms. As a member of the WNT pathway, orevious findings have also demonstrated that RET activates JNK *via* RAC in glial cells 41. Considering the important role of RET in HSCR, we suggested that the RAC isoforms have a strong association with HSCR. As for up‐stream regulators of the ARP2/3 complex, many studies have identified the WASP family as a macromolecule regulator of the ARP2/3 complex 42, 43. But on the other hand, very few studies have paid attention to those small molecules in the cell, such as miRNAs. The latter participate in multiple cellular processes, and are reported to be associated with many human diseases 44. According to bioinformatics predictions, we here propose a regulatory relationship between miR‐24‐1*, let‐7a* and the ARP2/3 complex. MiR‐24‐1* and let‐7a* have been studied in cancer and oncogenesis 45, 46, 47, but there is no evidence linking their function to HSCR. On the basis of such information, we propose a miR‐24‐1*/let‐7a*‐ARP2/3 complex‐RAC isoforms pathway in HSCR.

To further support the possible presence of this pathway, we determine the levels of the RAC isoforms in cases and control samples. The results demonstrated the both RAC1 and RAC2 were down‐regulated in HSCR, whereas RAC3 showed no difference, which suggested that RAC1 and RAC2 may be involved in the process of HSCR. In our study, three members of RAC isoforms presented different expression level in HSCR. According to previous studies 48, 49, RAC3 is mainly expressed in the central nervous system and is involved in the regulation of cell adhesion and nerve cell function. Therefore, we consider that in HSCR, non‐uniform expression levels of RAC1, RAC2 and RAC3 is caused by spatial specificity of protein expression. There is now a general consensus that down‐regulated RAC isoforms can restrain cells from migration and proliferation, for instance, the loss of RAC1 and RAC2 inhibits hematopoietic malignancies 50. In this study, 293T and SH‐SY5Y cell lines were selected as models for *in vitro* experiments. We transfected ARP2 and ARP3 siRNAs into both cell lines, and the results showed that RAC1 and RAC2 were down‐regulated, accompanied by the inhibition of migration and proliferation. With such findings, we speculated that the RAC isoforms are potential downstream targets of ARP2 and ARP3 and might play a significant role in the etiologic mechanism of HSCR. The cotransfected group was set to investigate a possible synergistic action of ARP2 and ARP3 siRNAs. Our data showed that RAC1 and RAC2 were not significantly down‐regulated compared with each single siRNA‐transfected groups. Meanwhile, as ARP2 and ARP3 were two subunits of a complex, we performed co‐IP experiments to ensure their association after inhibition by the corresponding siRNAs. The observations from this study indicates that a decrease in the levels of ARP2 or ARP3 could lead to a reduced association, which further support an involvement of the ARP2/3 complex.

As we transfected both siRNAs of RAC1 and RAC2 into both cell lines, the proliferation and migration were inhibited, without the synergistic action observed. To examine the initial status of miR‐24‐1* and let‐7a*, we first confirmed their expression levels in tissues samples, both of which were shown to be decreased in HSCR patients. Dual‐luciferase reporter assay was then performed to illustrate the potential regulatory relationships of miR‐24‐1*/ARP2 and let‐7a*/ARP3. *In vitro* experiments showed that increased levels of miR‐24‐1* and let‐7a* suppressed migration and proliferation, which also decreased ARP2, ARP3 and RAC isoforms at mRNA and protein levels without a noticeable synergistic action. We finally ascertained our assumption that the miR‐24‐1*/let‐7a*‐ARP2/3 complex‐RAC isoform pathway plays a role in HSCR.

**Figure 5:**
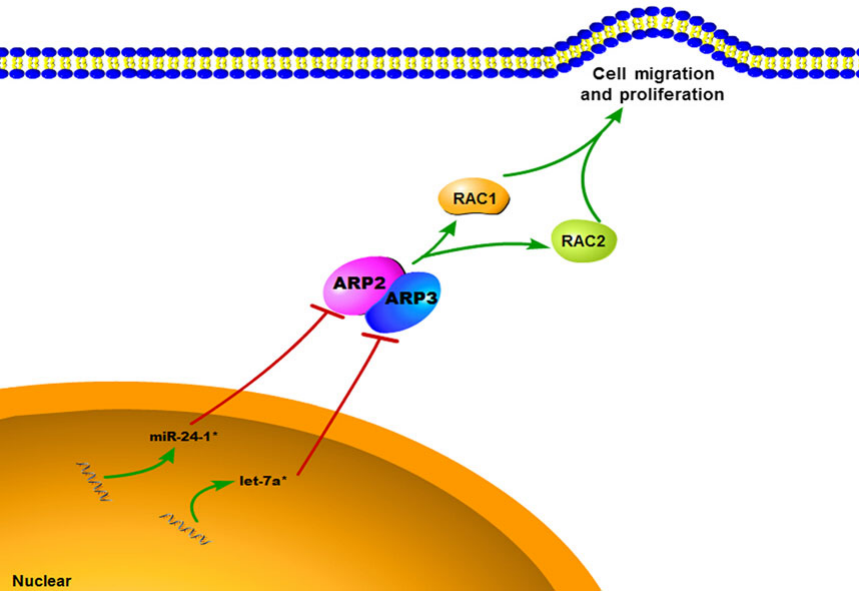
Schematic representation of the miR‐24‐1*/let‐7a*‐ARP2/3 complex‐RAC isoforms pathway in HSCR.

In conclusion, our study identified a novel pathway that is involved in the pathogenesis of HSCR. Some small molecular inhibitors of ARP2/3, miR‐24‐1* and let‐7a* regulate the heptamer. Meanwhile, the ARP2/3 complex suppresses migration and proliferation by down‐regulating RAC isoforms in aganglionosis (Fig. 5). Moreover, we discovered a novel regulatory mechanism of the ARP2/3 complex and RAC isoforms. However, for the studies on the mechanisms underlying HSCR, the best cell model is still ENCCs, and we are currently unable to verify the involvement of this pathway in such an ideal cell line. Nevertheless, this study suggests that miRNAs are involved in the regulation of the actin cytoskeleton by controlling the ARP2/3 complex and RAC signalling. Certainly more studies are needed to further dissect the cellular processes involved in HSCR development.

## Conflicts of interest

There are no conflicts of interest.

## Supporting information


**Figure S1** RNA oligos showed no influence on apoptosis and cell cycle.

**Figure S2** Transfection efficiency of siRNA‐ARP2 and siRNA‐ARP3.
