# Corrigendum

**DOI:** 10.1111/jcmm.13838

**Published:** 2018-09-25

In Tang et al,^1^ the original article contains incorrect Fig. 3A. The correct version of Figure 3 (Panel A) should have been as depicted below, all of the results and conclusions of the article remain unchanged.

**Figure 3:**
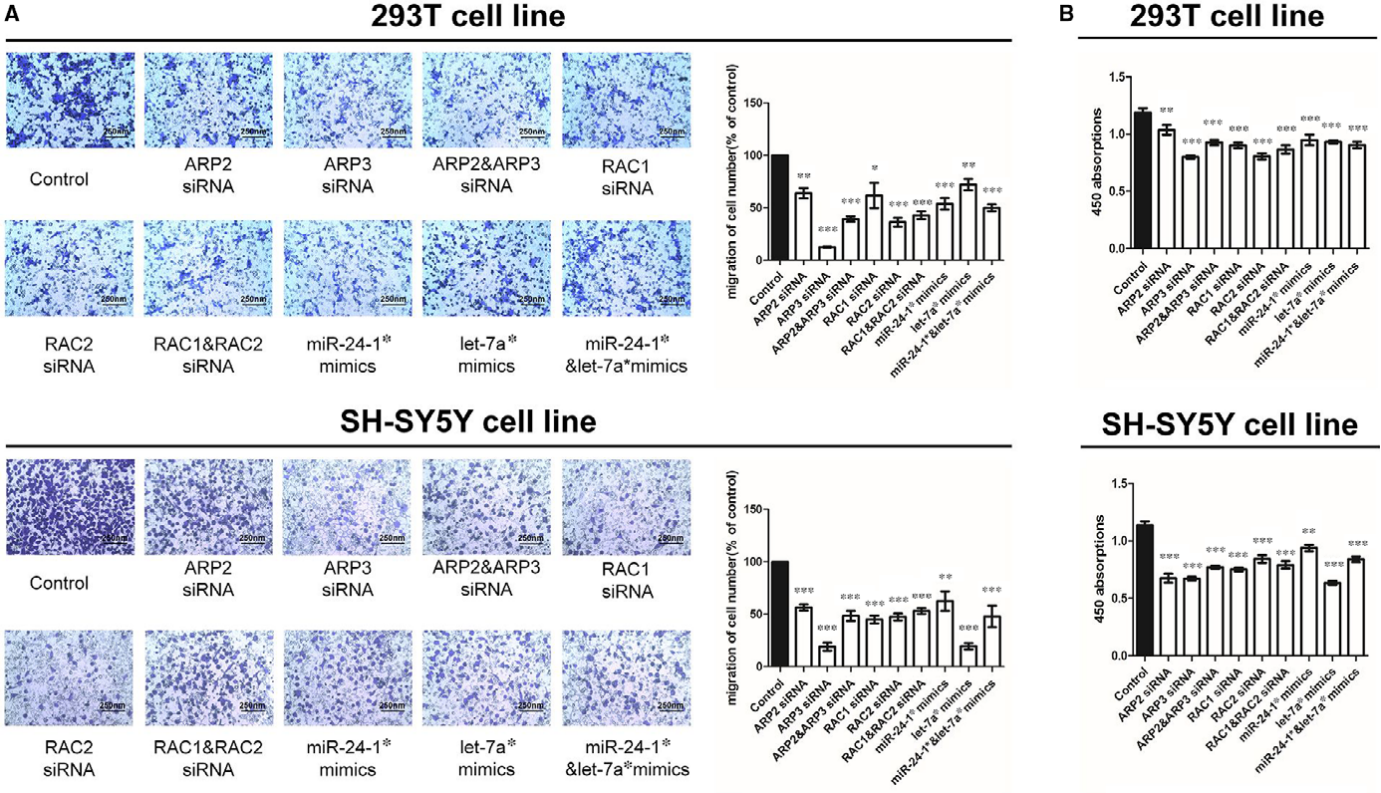
Cytobiology change after transfecting cell lines with RNA oligos. A, The representative images of metastasis cells at the bottom of the membrane stained with crystal violet were visualized as shown (left). The quantifications of cell migration were presented as percentage migrated cell numbers and the integrated intensity of migrated cells (right) (*P < 0.05, **P < 0.01, ***P < 0.001, n = 5, unpaired *t* test). B, The result of CCK‐8 assay of 450 nm absorption (*P < 0.05, **P < 0.01, ***P < 0.001, n = 6, unpaired *t* test). All tests were performed for three times and presented as mean ± SEM

The authors wished to apologize for any misunderstanding or inconvenience this may have caused.
