# Supplementary figures and images for: Suppressive action of miRNAs to ARP2/3 complex reduces cell migration and proliferation via RAC isoforms in Hirschsprung disease
Source: J Cell Mol Med. 2016 Mar 16;20(7):1266–75. doi: 10.1111/jcmm.12799 (PMC4929290; doi:10.1111/jcmm.12799)

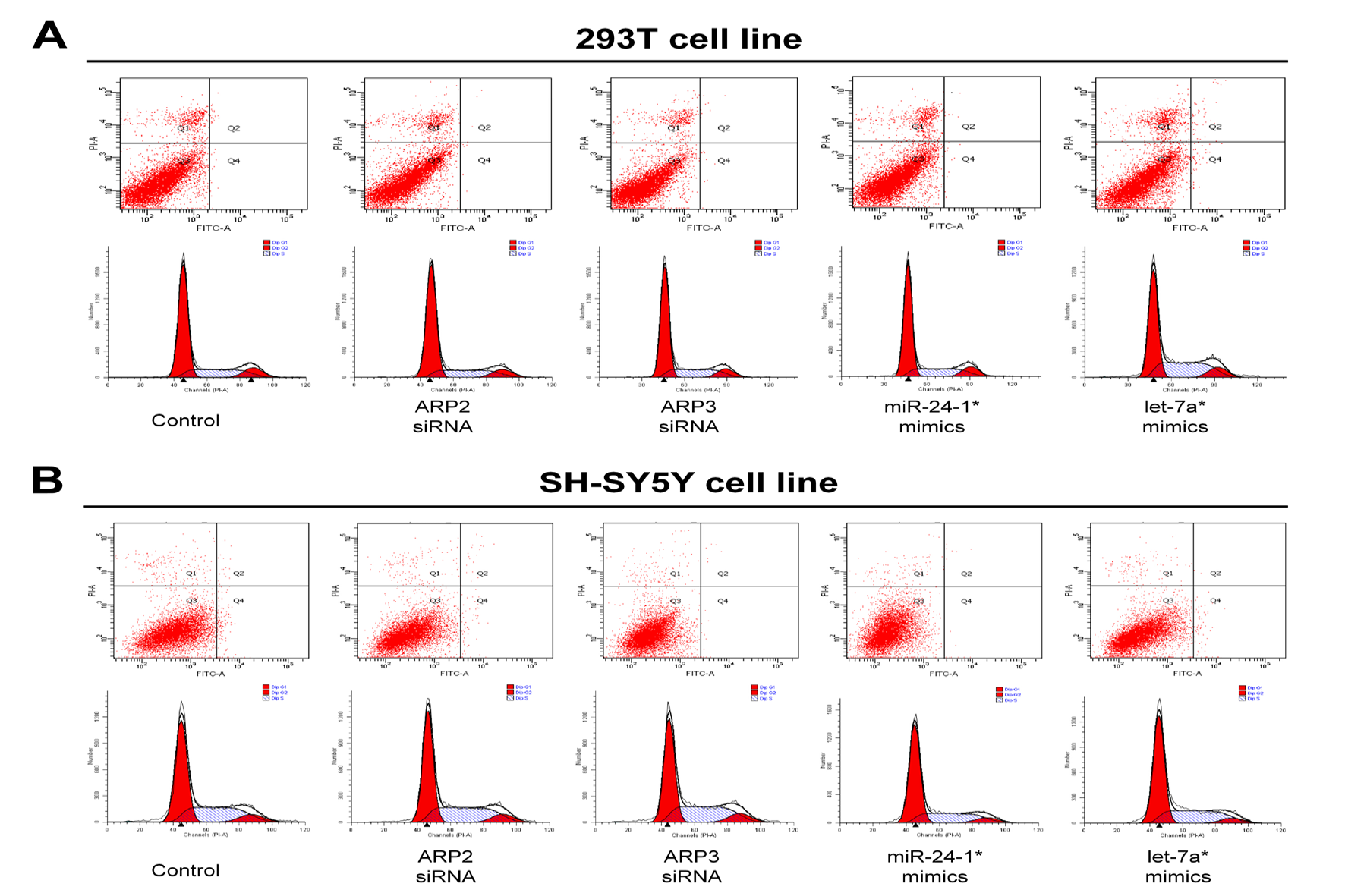

Supplement: Supplementary file 1 — Figure S1 RNA oligos showed no influence on apoptosis and cell cycle. [file JCMM-20-1266-s001.tif]

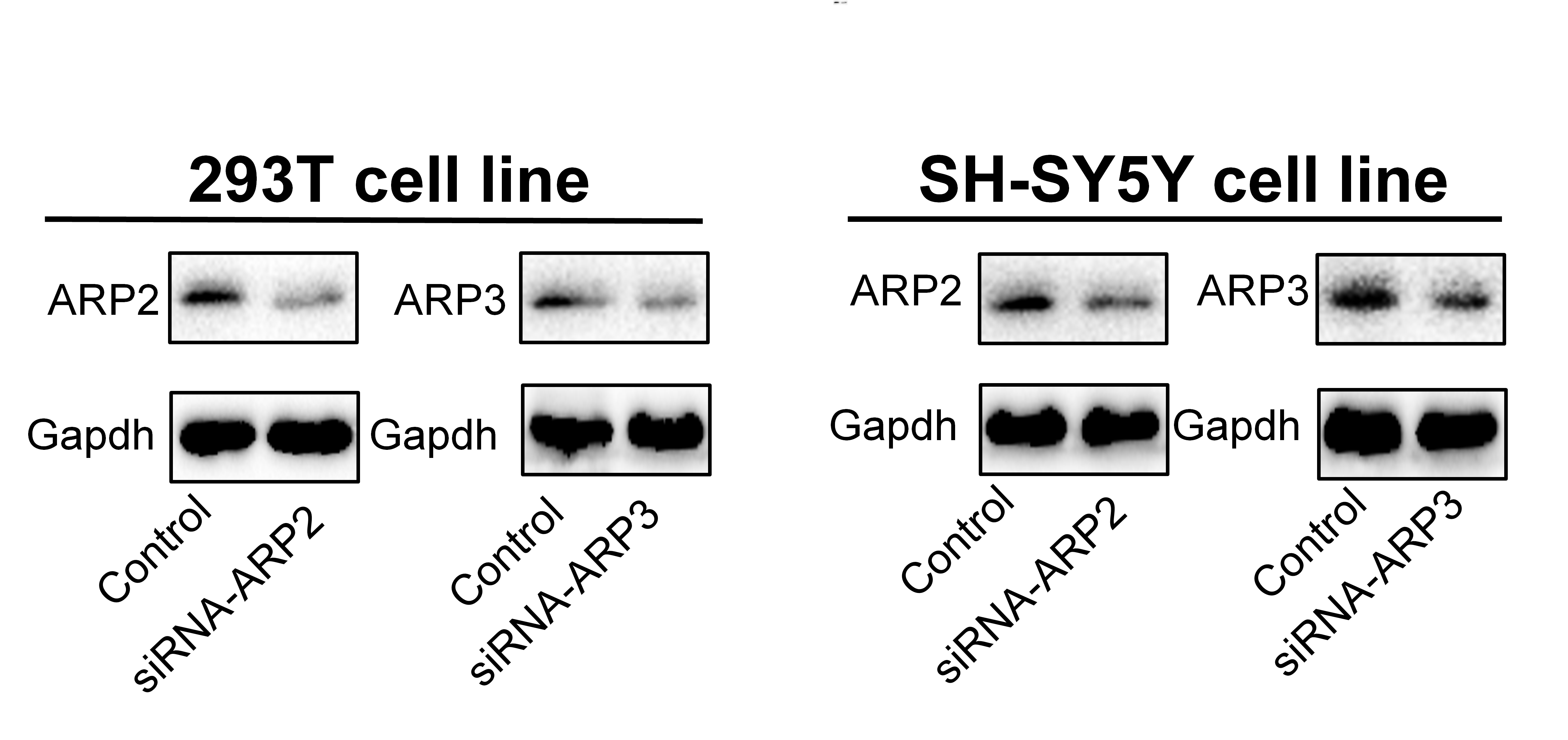

Supplement: Supplementary file 2 — Figure S2 Transfection efficiency of siRNA‐ARP2 and siRNA‐ARP3. [file JCMM-20-1266-s002.tif]
