# Supplementary material for: Suppressive action of miRNAs to ARP2/3 complex reduces cell migration and proliferation via RAC isoforms in Hirschsprung disease
Source: J Cell Mol Med. 2016 Mar 16;20(7):1266–75. doi: 10.1111/jcmm.12799 (PMC4929290; doi:10.1111/jcmm.12799)
Supplement: Supplementary file 3 [file JCMM-20-1266-s003.docx]

**Supplementary Figure. S1:**

**RNA oligos showed no influence on apoptosis and cell cycle.** (**A**): The results of apoptosis and cell cycle of siRNA-ARP2, siRNA-ARP3, miR-24-1* mimics and let-7a* treated groups in 293T cell lines. (**B**): The same works in SH-SY5Y cell line.

**Supplementary Figure. S2:**

**Transfection efficiency of siRNA-ARP2 and siRNA-ARP3.**
